# A Metabolomics Study of BPTES Altered Metabolism in Human Breast Cancer Cell Lines

**DOI:** 10.3389/fmolb.2018.00049

**Published:** 2018-05-15

**Authors:** G. A. Nagana Gowda, Gregory A. Barding, Jin Dai, Haiwei Gu, Daciana H. Margineantu, David M. Hockenbery, Daniel Raftery

**Affiliations:** ^1^Department of Anesthesiology and Pain Medicine, Northwest Metabolomics Research Center, University of Washington, Seattle, WA, United States; ^2^Fred Hutchinson Cancer Research Center, Seattle, WA, United States; ^3^Department of Chemistry, University of Washington, Seattle, WA, United States

**Keywords:** breast cancer, metabolomics, BPTES, MCF7, MDA-MB231, MCF10A, NMR, isotope tagging

## Abstract

The Warburg effect is a well-known phenomenon in cancer, but the glutamine addiction in which cancer cells utilize glutamine as an alternative source of energy is less well known. Recent efforts have focused on preventing cancer cell proliferation associated with glutamine addiction by targeting glutaminase using the inhibitor BPTES (bis-2-(5-phenylacetamido-1,3,4-thiadiazol-2-yl)ethyl sulfide). In the current study, an investigation of the BPTES induced changes in metabolism was made in two human breast cancer cell lines, MCF7 (an estrogen receptor dependent cell line) and MDA-MB231 (a triple negative cell line), relative to the non-cancerous cell line, MCF10A. NMR spectroscopy combined with a recently established smart-isotope tagging approach enabled quantitative analysis of 41 unique metabolites representing numerous metabolite classes including carbohydrates, amino acids, carboxylic acids and nucleotides. BPTES induced metabolism changes in the cancer cell lines were especially pronounced under hypoxic conditions with up to 1/3 of the metabolites altered significantly (*p* < 0.05) relative to untreated cells. The BPTES induced changes were more pronounced for MCF7 cells, with 14 metabolites altered significantly (*p* < 0.05) compared to seven for MDA-MB231. Analyses of the results indicate that BPTES affected numerous metabolic pathways including glycolysis, TCA cycle, nucleotide and amino acid metabolism in cancer. The distinct metabolic responses to BPTES treatment determined in the two breast cancer cell lines offer valuable metabolic information for the exploration of the therapeutic responses to breast cancer.

## Introduction

Breast cancer continues to have high incidence and is a major cause of death among women worldwide (Torre et al., 2016) (https://gco.iarc.fr/). Investigations focused on understanding the molecular origins and drivers of the disease have led to discovery of new gene expression patterns, which correlate with disease subtypes as well as the therapeutic outcome for breast cancer (Sørlie et al., 2001; van 't Veer et al., 2003; Paik et al., 2004). A number of recent investigations have focused on characterizing altered metabolism in cancer. In particular, the Warburg effect, which refers to the high rate of glycolysis observed in cancer cells even in the presence of oxygen (Warburg, 1956) is being re-examined in light of findings indicating a high dependence of cancer cells on glutamine. This so-called glutamine addiction is one of the major characteristics of the Warburg effect (DeBerardinis et al., 2007; Wise and Thompson, 2010), and has been shown to be of significant importance for mitochondrial metabolism, a major source of energy, and a source of nitrogen and carbon for biosynthesis (Gao et al., 2009; Wise and Thompson, 2010). Glutamine is converted to glutamic acid in mitochondria by the enzyme glutaminase. The glutamic acid thus formed fuels the TCA cycle through its conversion to alpha-ketoglutarate.

Recently, glutamine addiction in cancer cells has attracted major attention as a potential new therapeutic target for treating numerous types of cancers including breast cancer (Wang et al., 2010; Wise and Thompson, 2010; Katt and Cerione, 2014). Investigations using human breast cancer cell lines have shown that the oncogene, Myc, enhances the expression of glutaminase and thus increases glutamine metabolism in mitochondria (Gao et al., 2009; Wang et al., 2010). Inhibition of glutaminase is therefore sought as an important goal for preventing cancer cell proliferation, or killing cancer cells since the addiction may indicate a cell survival mechanism. A number of drugs have been developed as glutaminase inhibitors (Katt and Cerione, 2014); among these, BPTES (bis-2-(5-phenylacetamido-1,3,4-thiadiazol-2-yl)ethyl sulfide) is an important inhibitor that has been shown to be active in a variety of cancer cells. An advantage of BPTES is that it exhibits superior performance compared to the other related inhibitors (Katt and Cerione, 2014).

A number of investigations have focused on the understanding of the mechanism of inhibition of glutaminase activity by BPTES (Robinson et al., 2007; Hartwick and Curthoys, 2012; Thangavelu et al., 2012). Metabolomics offers a promising avenue for understanding the effect of overexpression of glutaminase or its inhibition, as the field has been very useful in identifying therapeutic targets and a number of disease biomarkers (Nagana Gowda and Raftery, 2013). To date, only a very few metabolite profiling studies have investigated the metabolism of glutamine-addicted cells or the effect of BPTES on metabolism. One such study investigated cancer cell metabolism under normoxic and hypoxic conditions with or without BPTES using a human lymphoma cell line. In this study, it was shown that increased levels of TCA cycle metabolites were observed under hypoxic conditions and glutaminase inhibition by BPTES caused cell death (Le et al., 2012). More recently, based on global metabolite profiling of tumors as well as numerous human breast cancer cell lines including MCF7 and MDA-MB231, glutaminase overexpression was shown to elevate the levels of 2-hydroxyglutarate, which potentially serves as an oncometabolite biomarker for some types of breast cancer (Terunuma et al., 2014). However, currently, there are no investigations focused on altered metabolism induced by BPTES in breast cancer.

In the current study, focused on better understanding BPTES-induced metabolic changes in breast cancer, we have investigated metabolite profiles of two human breast cancer cell lines, MCF7 and MDA-MB231 along with a non-cancerous cell line, MCF10A, combining ^1^H 1D NMR and isotope tagged ^1^H-^15^N 2D NMR spectroscopy (Tayyari et al., 2013). Isotope tagging improves the resolution of NMR experiments for improved metabolite profiling. The three cell types were grown using identical conditions under normoxia or hypoxia, and with or without treatment by BPTES. It is shown that the effect of BPTES on metabolism was pronounced under hypoxia for both cancer cell lines. Further, between the two cancer cell lines, the BPTES induced effect was substantially higher for MCF7 cells compared to MDA-MB231 cells. After BPTES treatment twice as many detected metabolites were altered significantly in MCF7 compared to MDA-MB231 cells, and a number of metabolic pathways were affected. To the best of our knowledge, this is the first metabolomics study that investigates altered metabolism in breast cancer cells induced by the glutaminase inhibitor, BPTES. Identification of the altered levels of metabolites potentially offers valuable metabolite biomarkers for exploration of responses to therapy in the treatment of breast cancer.

## Materials and methods

BPTES (bis-2-(5-phenylacetamido-1,3,4-thiadiazol-2-yl)ethyl sulfide), (2-bromoethyl) trimethylammonium bromide and 3-(Trimethylsilyl)propionic acid-2,2,3,3-d_4_ sodium salt (TSP) were obtained from Sigma-Aldrich (St. Louis, MO). 4-(4,6-dimethoxy[1,3,5]triazin-2-yl)-4-methylmorpholinium chloride (DMTMM) was obtained from Acros Organic (Pittsburgh, PA), while ^15^N-phthalimide potassium was obtained from Cambridge Isotope Laboratories (Andover, MA). Human breast cancer cell lines MCF7 and MDA-MB231, and the non-cancerous cell line, MCF10A, were procured from the Hockenbery lab at the Fred Hutchinson Cancer Research Center. All chemicals and solvents used were of analytical grade and used without further purification.

### Cell culture

MCF7 and MDA-MB231 cells were cultured in DMEM medium (Gibco, Los Angeles, CA USA) containing 10% fetal calf serum, 2mM glutamine, 1% penicillin-streptomycin (Gibco, Grand Island, NY, USA) at 37°C with 5% CO_2_. MCF10A cells were grown in complete growth medium, 1:1 mixture of Dulbecco's Modified Eagle's Medium and Ham's F12 Medium (Gibco, Grand Island, NY) supplemented with 20 ng/mL epidermal growth factor (EGF), 100 ng/mL cholera toxin, 10 μg/mL insulin, 500 ng/mL hydrocortisone (Sigma-Aldrich St. Louis, MO, USA), 5% horse serum, and 1% penicillin-streptomycin. The cells were plated at a density of 5 × 10^6^ on 150 × 25 mm tissue culture dishes (Corning Incorporated, MA) in 25 mL complete growth medium. After 18 h, fresh medium was replaced. A total of 12 plates were grown for each cell line so that each study group had three replicates (Table S1). For each cell type, six plates were incubated under normoxic conditions (21% O_2_, 5% CO_2_, 37°C) and the other six plates were incubated under hypoxic conditions (2% O_2_, 5% CO_2_, 37°C). Half of the cell plates from normoxic (*n* = 3) and hypoxic (*n* = 3) conditions were treated with 20 μM BPTES inhibitor (Sigma-Aldrich St. Louis, MO) before incubation.

### Metabolite extraction

After 24 h incubation, the cell media were removed and the cells washed with 30 mL cold water; a mixture of methanol/chloroform (9.5 mL; 9:1 v/v) was then immediately added to the plates to quench the cells and extract metabolites. Cell lysates were obtained by keeping the plates at −75°C for 5 min and thawing them at room temperature. Cell remnants were scraped from the culture dishes and collected in fresh tubes along with the cell lysates. Resulting mixtures were centrifuged at 13,000 rpm for 5 min and supernatant solutions that contained cell metabolites were transferred to fresh tubes and dried overnight using a Speedvac at 30°C. The dried residues were dissolved in 600 μL 0.1 M phosphate buffer (pH 7.4) in D_2_O solvent containing 50 μM TSP and the solutions transferred to 5 mm NMR tubes for metabolite analysis using ^1^H 1D NMR spectroscopy.

### Metabolite labeling with a ^15^N- isotope tag

**Figure 1:**
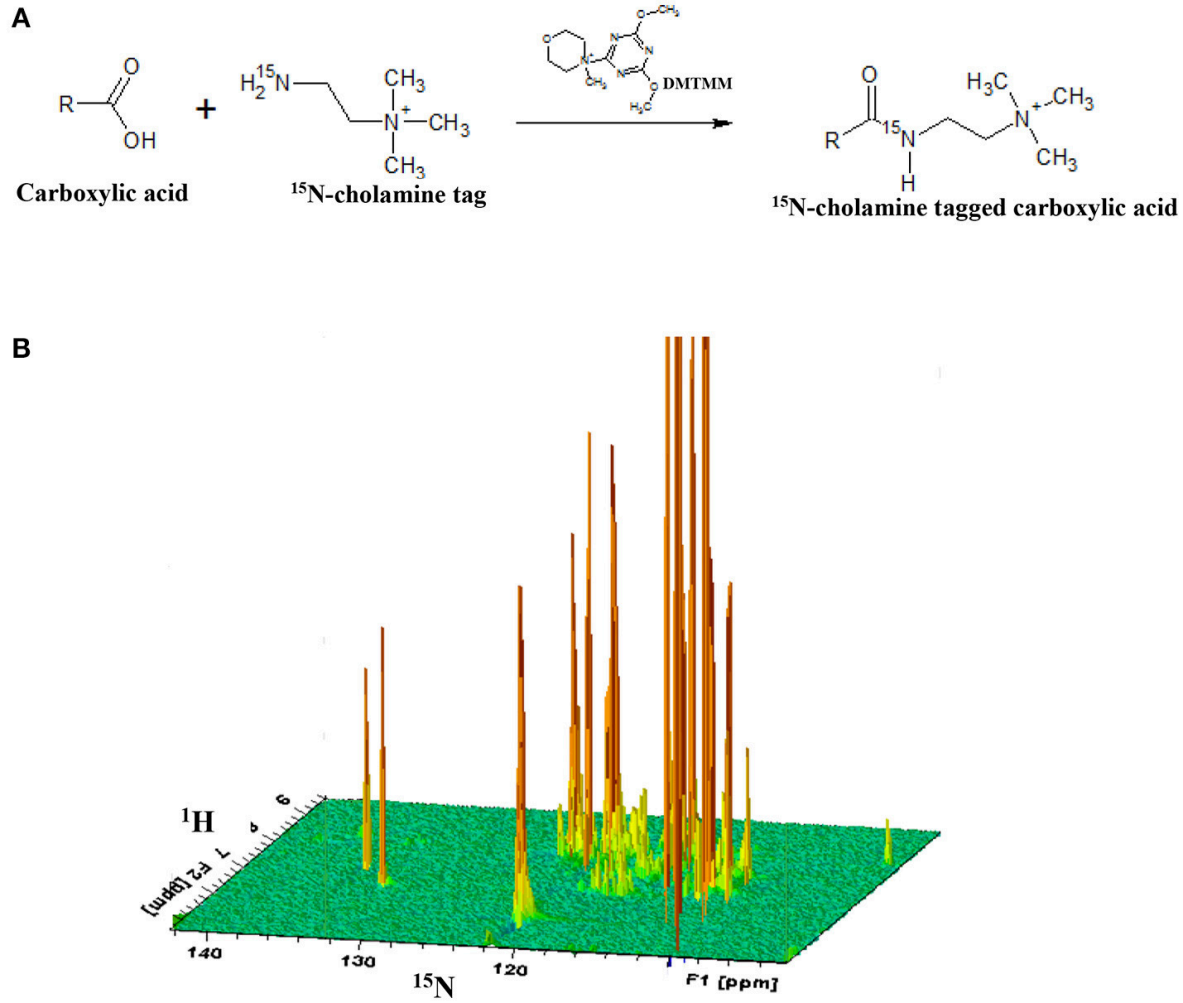
**(A)** General reaction for tagging of carboxylic group containing metabolites with ^15^N-cholamine tag; **(B)** schematic 3D view of a typical 2D ^1^H-^15^N HSQC NMR spectrum of a sample with ^15^N-cholamine tagging of carboxylic acid containing metabolites. DMTMM:4-(4,6-dimethoxy[1,3,5]triazin-2-yl)-4-methylmorpholinium chloride.

After acquiring ^1^H 1D NMR spectra as described below for cell extracts, the solutions were dried and reconstituted in 550 μL water. Carboxyl group containing metabolites were then labeled with ^15^N-cholamine (Figure 1), which was synthesized using a two-step reaction following the protocol described previously by our laboratory (Tayyari et al., 2013). Briefly, ^15^N-cholamine (5 mg, 50 μmol) was added to solutions of cell extracts in Eppendorf tubes and pH adjusted to 7.0 with 1 M hydrochloric acid (HCl) or sodium hydroxide (NaOH). DMTMM (15 mg) was then added as a catalyst to help initiate the reaction, and the mixtures were then stirred at room temperature for 4 h to complete the reaction. The resulting solutions were mixed with a small volume (25 μL) of D_2_O for NMR field-frequency locking. To maintain amide protonation the pH was adjusted to 5.0 by adding 1 N HCl or 1 N NaOH. The solutions were then transferred to 5 mm NMR tubes for detection of the isotope tagged metabolites using two-dimensional NMR spectroscopy.

### NMR spectroscopy

All NMR experiments were performed at 298 K on a Bruker Avance III 800 MHz spectrometer equipped with a cryoprobe and Z-gradients. Before labeling with the cholamine tag, ^1^H 1D NMR experiments were performed on the cell extracts using the CPMG (Carr-Purcell-Meiboom-Gill) pulse sequence with residual water signal suppression using presaturation. A spectral width of 9,615 Hz, time domain points of 32 K, a recycle delay of 6 s, 16 dummy scans and 64 scans were used. The raw data were then Fourier transformed after multiplying with an exponential window function using a line broadening (LB) of 0.5 Hz and spectrum size of 32 K points. Resulting 1D spectra were phase and baseline corrected. To detect the carboxyl group containing metabolites after isotope tagging, sensitivity-enhanced ^1^H-^15^N 2D HSQC experiments were performed with an INEPT transfer delay of 6 ms corresponding to the ^1^J_NH_ coupling of 90 Hz. Spectral widths for the ^1^H and ^15^N dimensions were approximately 8 and 3 kHz, respectively. One hundred and twenty-eight free induction decays of 1,024 data points each were collected in the indirect dimension (t_1_) with 16 transients per increment. ^15^N decoupling during the direct acquisition dimension (t_2_) was achieved with the GARP (Globally Optimized Alternating-Phase Rectangular Pulses) sequence. The resulting 2D data were zero-filled to 2,048 points in the t_2_ and 1,024 in the t_1_ dimension after forward linear prediction to 256 points. A 90° shifted squared sine-bell window function was applied to both dimensions before Fourier transformation. Chemical shifts were referenced to the TSP signal for ^1^H 1D NMR or the derivatized formic acid signal (^1^H: 8.05 ppm; ^15^N: 123.93 ppm) in the 2D HSQC NMR spectra for isotope tagged samples.

### Data analyses

Bruker Topspin versions 3.0 or 3.1 and the Bruker AMIX software package were used for NMR data processing and analyses. Metabolites were identified based on established chemical shift databases (Tayyari et al., 2013; Wishart et al., 2013; Nagana Gowda et al., 2015) and quantitated after normalizing the spectra to their total sums. For 1D NMR spectra, integrals for characteristic and well-resolved metabolite peaks were used to obtain the relative metabolite concentrations. For 2D spectra, Bruker Topspin 2D peak integration was used to obtain the relative metabolite concentrations of isotope tagged metabolites. The metabolite profiles were analyzed combining univariate and multivariate statistical methods. Multivariate hierarchical cluster analysis (HCA) was used to enable global visualization of natural clusters and altered metabolite profiles between different groups of cells based on comparing distances between pairs of samples. Pearson correlations between metabolites in each cell type under normoxia, hypoxia and with BPTES treatment were also calculated. Individual metabolite differences between pairs of cell treatments, were analyzed using the Student's *t*-test and fold changes. Metabolite changes that exhibited *p* < 0.05 were considered significant. HCA and correlation analysis were performed using R statistical software (version 2.12.2).

## Results

NMR experiments at 800 MHz provided highly resolved spectra for all cell extracts. 1D NMR experiments enabled identification and quantitation of a total of 32 intracellular metabolites, while 2D NMR experiments that targeted carboxylic group containing metabolites in the same cells based on selective labeling using an ^15^N-cholamine tag enabled identification of a total of 19 metabolites. A total of 41 unique metabolites were obtained from the combination of 1D and 2D NMR approaches. Of these, 9 metabolites namely arginine, carnitine, citrate, *p*-coumaric acid, N-acetylglycine, malate, pyroglutamate, oxalic acid and succinate were unique to the 2D NMR method. Table 1 lists metabolites identified by both 1D and 2D NMR; all metabolites identified by 2D NMR are marked in the table. Although the relative concentrations for the metabolites identified by both 1D and 2D NMR were comparable, in this study all metabolites derived from 1D NMR and the 9 unique metabolites derived from 2D NMR were used in the analysis and for reporting.

**Table 1:** Fold change *(F)* and *p*-value *(P)* for metabolites between normoxia and hypoxia and between BPTES treated and untreated MCF10A, MCF7, and MDA-MB231 cells.

		**No BPTES: Normoxia vs. Hypoxia**						**Normoxia cells: with vs. without BPTES**						**Hypoxia cells: with vs. without BPTES**					
**Number**	**Metabolite**	**MCF10A**		**MCF7**		**MDA-MB231**		**MCF10A**		**MCF7**		**MDA-MB231**		**MCF10A**		**MCF7**		**MDA-MB231**	
		***F***	***P***	***F***	***P***	***F***	***P***	***F***	***P***	***F***	***P***	***F***	***P***	***F***	***P***	***F***	***P***	***F***	***P***
1	Acetic acid^&^	1.0	0.7040	1.3	0.093899	1.2	0.04072	1.2	0.051	1.1	0.527	1.0	0.735	1.1	0.212	1.3	0.021	1.1	0.3587
2	Alanine^&^	2.1	0.0155	1.7	0.000029	0.8	0.00014	0.9	0.749	0.9	0.373	1.0	0.343	1.0	0.813	0.9	0.001	0.9	0.0528
3	Arginine^*^	1.1	0.5506	1.2	0.370822	1.0	0.94351	1.1	0.611	0.9	0.648	1.0	0.668	1.2	0.126	0.9	0.459	1.0	0.7457
4	Asparagine	0.7	0.1891	0.9	0.116774	0.8	0.46650	1.5	0.181	1.0	0.650	1.2	0.303	0.9	0.578	1.0	0.570	0.9	0.8023
5	Aspartic acid	0.2	0.0006	0.7	0.012818	0.5	0.00580	0.8	0.117	0.9	0.293	0.9	0.395	1.0	0.762	0.9	0.627	1.2	0.2208
6	AXP	0.5	0.0238	1.0	0.813836	0.9	0.33243	0.8	0.170	0.8	0.078	0.8	0.202	1.0	0.910	0.6	0.014	1.3	0.3152
7	Carnitine^*^	0.8	0.0313	1.1	0.659678	0.9	0.31986	0.9	0.001	1.6	0.009	0.7	0.145	0.7	0.099	1.2	0.529	1.1	0.4407
8	Citrate^*^	1.6	0.0125	0.8	0.099086	0.7	0.02499	1.0	0.989	1.2	0.159	0.9	0.607	1.0	0.831	1.1	0.705	1.5	0.0047
9	Creatine	0.9	0.2095	1.0	0.839392	1.1	0.04297	1.0	0.764	1.0	0.684	1.0	0.684	1.0	0.530	1.0	0.291	0.9	0.0136
10	Formic acid^&^	1.0	0.8875	1.5	0.040065	1.1	0.48513	1.0	0.862	1.2	0.372	1.0	0.701	0.8	0.469	1.3	0.013	0.8	0.2341
11	Fumaric acid^&^	NA	0.2302	0.3	0.512225	0.8	0.13178	NA	0.345	1.5	0.629	1.1	0.664	1.0	1.000	0.5	0.887	0.6	0.0341
12	Glucose	1.8	0.0006	1.3	0.135911	0.8	0.62071	1.4	0.057	0.9	0.815	0.5	0.330	0.9	0.247	0.7	0.027	1.4	0.3846
13	Glutamic acid	0.3	0.0002	0.6	0.002484	0.8	0.00711	0.9	0.211	0.9	0.154	1.0	0.967	1.1	0.599	0.8	0.002	1.1	0.1410
14	Glutamine	0.6	0.0065	1.1	0.193823	0.9	0.47859	1.1	0.111	1.0	0.895	1.1	0.074	1.1	0.173	1.0	0.883	1.0	0.8187
15	Glutathione^&^	0.5	0.0006	0.7	0.014849	0.7	0.01350	0.8	0.017	1.0	0.519	1.0	0.910	0.9	0.371	0.9	0.285	1.1	0.2561
16	Glycerophosphocholine	0.8	0.0019	0.6	0.001856	0.7	0.00008	0.9	0.001	1.0	0.595	0.9	0.199	1.2	0.041	1.0	0.562	1.3	0.0001
17	Glycine^&^	0.4	0.0052	0.9	0.137208	0.7	0.00029	1.1	0.304	0.9	0.553	1.0	0.901	1.2	0.092	0.9	0.010	1.0	0.4467
18	Glycine-N-acetyl^*^	1.1	0.2659	0.8	0.028745	0.8	0.18737	0.8	0.074	0.9	0.215	1.0	0.955	0.8	0.001	0.8	0.002	1.0	0.8173
19	3-Methyl-2-oxovalerate	2.0	0.7078	1.0	0.902518	1.0	0.84580	3.2	0.465	1.0	0.991	1.0	0.885	1.0	1.000	1.3	0.045	0.8	0.1094
20	Isoleucine^&^	3.9	0.0023	1.4	0.000138	1.0	0.24736	1.4	0.520	1.0	0.888	1.0	0.883	0.7	0.029	1.0	0.411	0.9	0.1042
21	Lactic acid^&^	3.2	0.0063	1.2	0.207556	1.8	0.02774	0.9	0.462	1.1	0.446	1.2	0.584	0.8	0.273	1.1	0.459	0.6	0.0182
22	Leucine	2.1	0.0011	1.4	0.000054	1.0	0.17853	1.0	0.954	1.0	0.608	1.0	0.721	0.8	0.049	1.0	0.755	0.9	0.1196
23	Lysine	1.0	0.7933	1.2	0.146758	1.2	0.04425	1.2	0.053	1.1	0.709	1.0	0.699	1.0	0.844	1.1	0.162	0.8	0.0419
24	Malate^*^	1.5	0.2426	1.1	0.879136	0.8	0.29070	1.3	0.260	1.0	0.935	1.0	0.678	1.3	0.271	1.5	0.236	1.1	0.5995
25	Myoinositol	0.7	0.0158	0.8	0.016440	0.9	0.10066	0.7	0.015	0.9	0.227	1.0	0.907	0.8	0.289	0.9	0.102	1.1	0.2231
26	NAD	1.1	0.4557	0.3	0.049526	0.8	0.40443	1.2	0.022	0.7	0.146	0.9	0.623	1.0	0.759	1.1	0.927	0.8	0.4657
27	NADP	NA	0.0835	NA	0.218172	1.0	1.00000	3.5	0.427	NA	0.666	1.0	0.653	NA	0.189	NA	0.049	0.8	0.4746
28	Oxalic acid^*^	1.3	0.1496	1.1	0.289234	0.9	0.61455	1.0	0.943	1.2	0.327	1.0	0.525	1.1	0.544	1.4	0.011	1.1	0.3073
29	p-coumaric acid^*^	0.6	0.1167	1.1	0.485216	0.7	0.17160	1.0	0.912	1.1	0.753	0.9	0.520	1.7	0.022	0.9	0.578	1.1	0.7341
30	Phenylalanine^&^	0.9	0.2000	1.2	0.002376	1.0	0.73171	1.2	0.031	1.0	0.439	1.0	0.868	1.2	0.025	1.0	0.019	0.9	0.0695
31	Phosphocholine	0.4	0.0034	5.3	0.000003	1.1	0.10850	0.8	0.061	1.2	0.065	1.0	0.992	0.8	0.133	0.8	0.025	1.2	0.0440
32	Phosphocreatine	0.6	0.0016	0.9	0.092105	0.9	0.03617	0.8	0.012	1.0	0.474	1.1	0.461	1.1	0.399	0.9	0.417	1.2	0.2431
33	Proline	1.4	0.0594	0.8	0.018700	0.8	0.03494	1.0	0.747	1.0	0.550	1.0	0.557	1.1	0.205	1.0	0.637	1.1	0.1074
34	Pyroglutamate^*^	0.6	0.0004	0.9	0.012237	1.0	0.92589	1.0	0.194	1.0	0.843	1.0	0.098	1.0	0.503	1.0	0.700	1.0	0.6827
35	Succinate^*^	1.0	0.8575	1.2	0.101180	1.0	0.42919	1.4	0.008	1.1	0.324	1.1	0.209	1.0	0.780	1.0	0.962	0.9	0.1043
36	Threonine^&^	0.7	0.0005	0.9	0.026454	0.8	0.02308	0.9	0.231	1.0	0.414	1.0	0.619	1.0	0.702	0.9	0.037	1.0	0.7084
37	Tryptophan	NA	0.1688	1.2	0.185712	1.1	0.71552	NA	0.610	1.2	0.122	1.0	0.794	NA	0.150	1.2	0.075	0.8	0.2313
38	Tyrosine	0.9	0.2082	1.3	0.002780	1.0	0.57860	1.3	0.049	1.0	0.439	1.0	0.983	1.2	0.025	1.0	0.371	0.9	0.0470
39	Uridine	0.6	0.0019	0.8	0.026587	0.7	0.00263	0.8	0.015	0.9	0.190	0.8	0.008	0.8	0.131	0.8	0.018	1.3	0.0829
40	UXP	0.5	0.0013	0.6	0.005057	0.6	0.00055	0.8	0.011	0.9	0.327	0.8	0.022	0.8	0.186	0.8	0.023	1.6	0.0916
41	Valine	1.3	0.0658	1.4	0.001058	1.1	0.05146	0.9	0.387	1.0	0.496	1.0	0.955	0.8	0.105	1.0	0.114	0.9	0.0763

*NA, not available as the metabolite level was too low or not detected in one or both groups*.

* Metabolites identified using 2D NMR;

& *Metabolites identified using both 1D and 2D NMR but quantitated using 1D NMR*.

### Cancer vs. non-cancerous cells

Metabolite profiles of cancer cells, MCF7 and MDA-MB231, were distinctly different from each other and from non-cancerous cells, MCF10A. Levels of a majority of the metabolites were significantly different between cancer and non-cancer cells. The number of metabolites that were altered significantly (*p* < 0.05) was 25 between MCF7 and MCF-10A, 29 between MDA-MB231 and MCF-10A, and 27 between MCF7 and MDA-MB231 (Table S2). Overall, the differences in metabolite levels between cancer and non-cancerous cells varied by up to two orders of magnitude, while the differences between the two breast cancer cells varied by as much as twenty-fold. The significantly altered metabolites between cancer and non-cancerous cells represented numerous pathways including glycolysis, TCA cycle, amino acid and nucleotide metabolisms.

The Warburg effect was quite evident in both cancer cell types as glucose levels dropped by a factor of 10 when compared to the non-cancerous cells, signifying the high rate of glucose metabolism (Table S2). Further, the high glucose metabolism was more pronounced for MCF7 cells (*p* < 0.00003) than MDA-MB231 (*p* < 0.0001). High levels of lactate were also observed for both cancer cell types, increasing by a factor of 3.8 for MCF7 cells and 3.5 for MDA-MB231. Further, in accordance with the difference in the rate of glycolysis, lactate production was more pronounced for MCF7 cells (*p* < 0.002) than MDA-MB231 (*p* < 0.01).

### Hypoxia induced metabolite changes to cells

**Figure 2:**
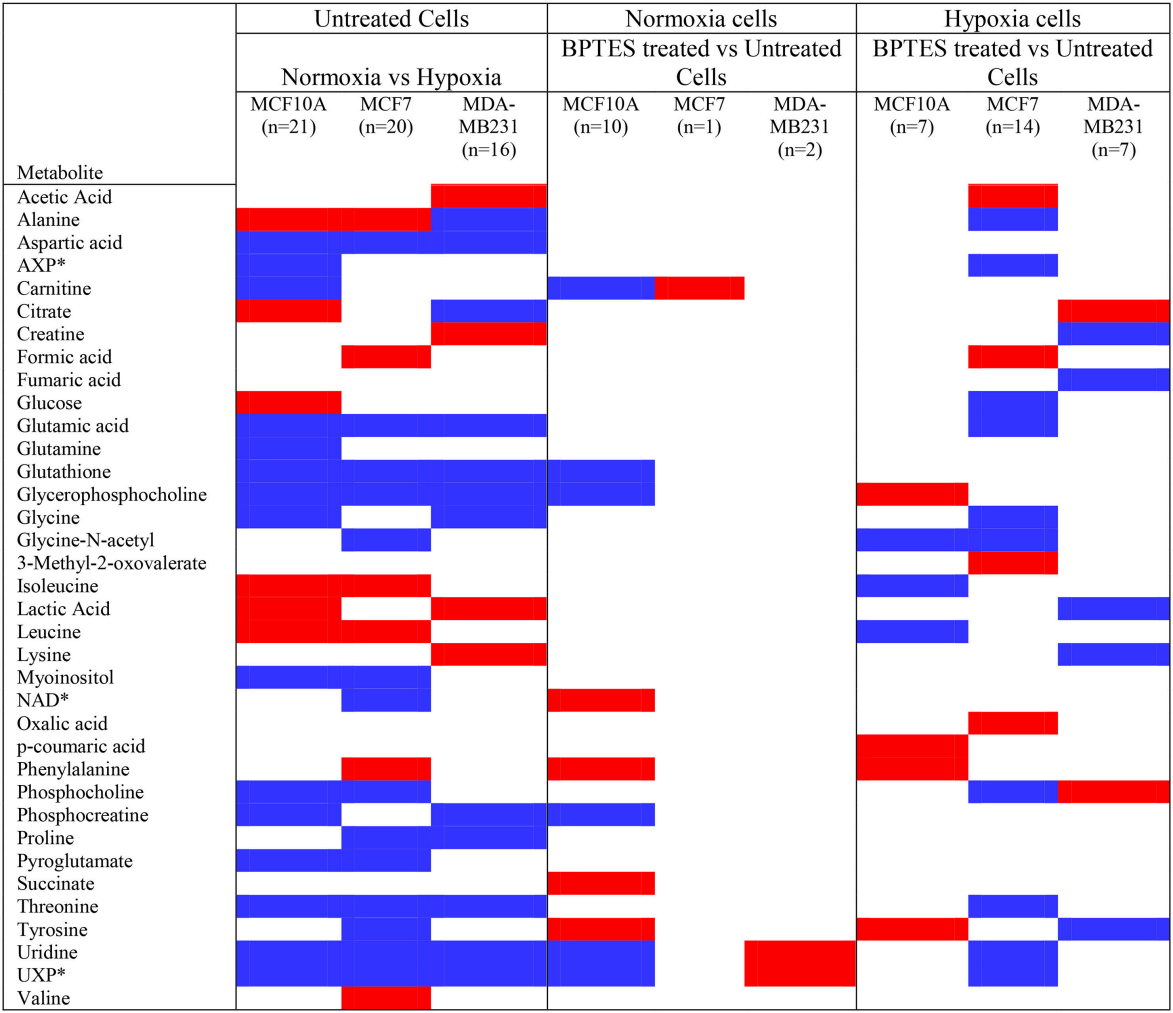
Metabolites that increased or decreased significantly (*p* < 0.05) in hypoxia cells vs. normoxia cells (MCF10A, MCF7, and MDA-MB231) as well as in BPTES treated vs. untreated cells (for interpretation of the references to color in the description of this figure in the text, the reader is referred to the Web version of this article). Red: increase in hypoxia compared to normoxia cells or BPTES treated cells compared to untreated cells; Blue: decrease in hypoxia compared to normoxia cells or BPTES treated cells compared to untreated cells. ^*^AXP is a combination of AMP (adenosine monophosphate), ADP (adenosine diphosphate), and ATP (adenosine triphosphate); NAD: Nicotinamide adenine dinucleotide, oxidized; UXP is a combination of UMP (uridine monophosphate), UDP (uridine diphosphate), and UTP (uridine triphosphate).

A significant effect of hypoxia on the cancer and non-cancerous cells was evident from the altered levels for a large number of metabolites. The effect, however, was different for each cell type. For MCF7 cells, 20 metabolites were altered significantly (*p* < 0.05) due to hypoxia compared to normoxia; of these, 6 metabolites were upregulated and 14 metabolites were downregulated compared to normoxic conditions (Figure 2; Table 1). For MDA-MB231 cells, 16 metabolites were altered significantly (*p* < 0.05) due to hypoxia; of these, 4 metabolites were upregulated and 12 metabolites were downregulated relative to normoxia. The hypoxic effect was even more pronounced for the non-cancerous cells, MCF-10A; a total of 21 metabolites were altered significantly with 6 metabolites upregulated and 15 metabolites downregulated. Hypoxia altered metabolite levels by more than three-fold in some cases, with majority of them decreased relative to normoxia cells (Figure 2; Table 1).

**Figure 3:**
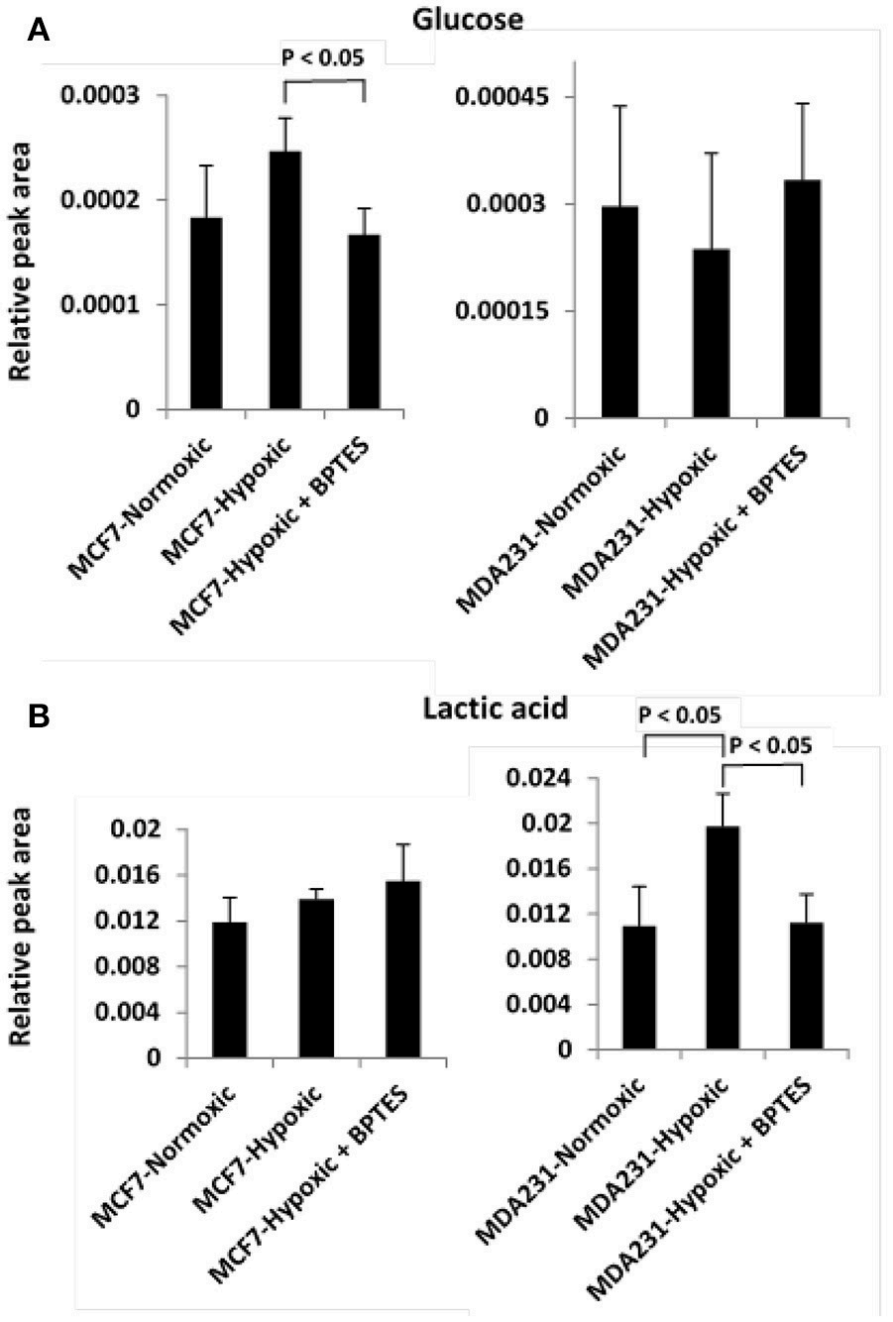
Variation of levels of two metabolites associated with the glycolysis pathway **(A)** glucose and **(B)** lactic acid in the two cancer cell lines, MCF7 and MDA-MB231 under the conditions of normoxia, hypoxia and hypoxia treated with the glutaminase inhibitor, BPTES.

**Figure 4:**
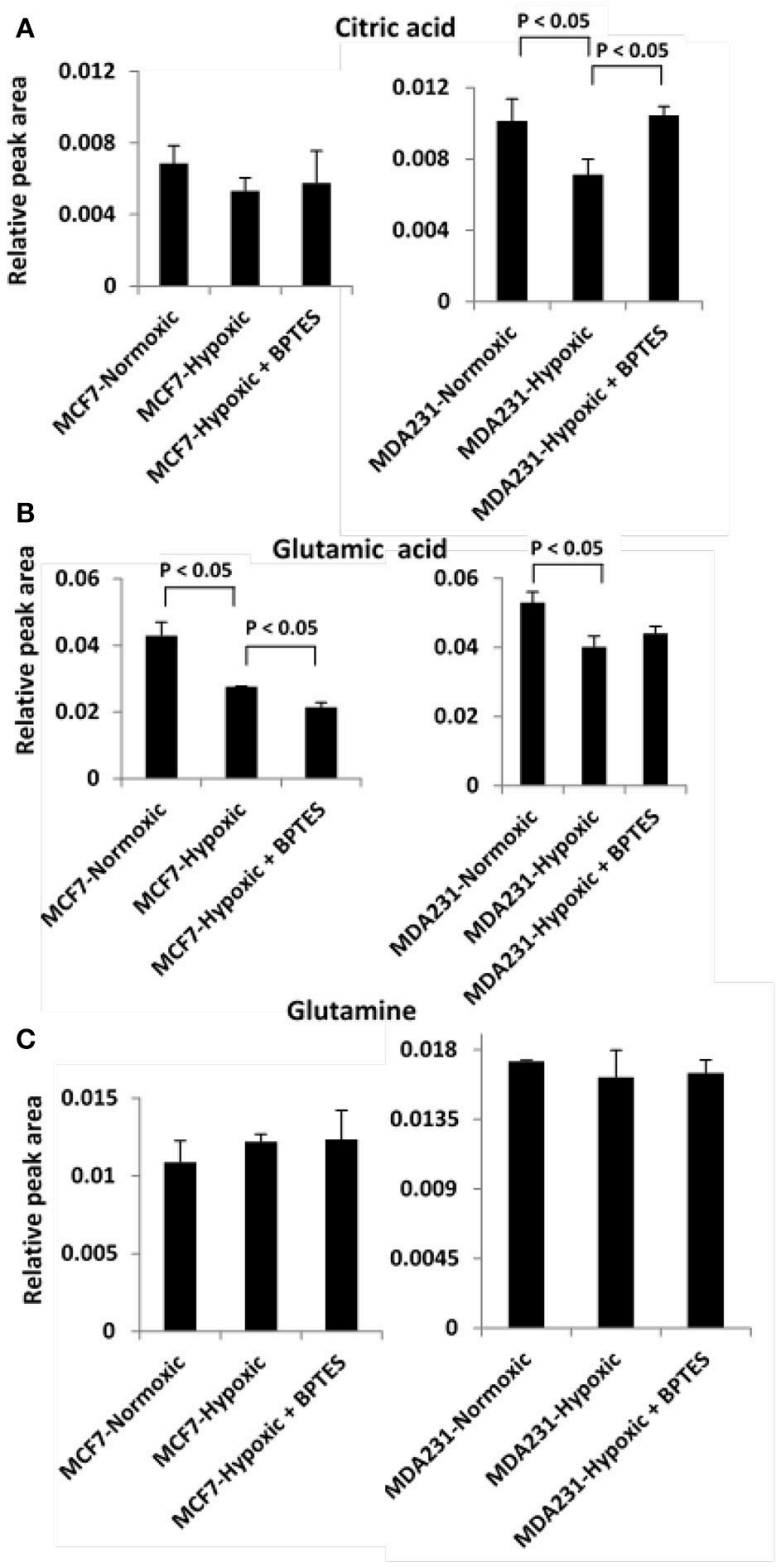
Variation of levels of three metabolites associated with TCA cycle and glutamine addiction to cancer cells **(A)** citric acid; **(B)** glutamic acid, and **(C)** glutamine in the two cancer cell lines, MCF7 and MDA-MB231 under the conditions of normoxia, hypoxia and hypoxia treated with the glutaminase inhibitor, BPTES.

Hypoxia affected glycolysis and TCA cycle metabolism for both cancer cell lines, albeit differently (Figures 3, 4). Specifically, hypoxia caused increased levels for both glucose and lactate in MCF7, while in MDA-MB231 cells glucose decreased and lactate increased. However, none of these changes was significant except for the increased level of lactate in MDA-MB231 cells. Glutamic acid levels, which are closely related to activity of the TCA cycle, were significantly altered. Glutamic acid decreased in MCF7 cells, while in MDA-MB231 cells both citrate and glutamic acid decreased.

### Effect of BPTES on metabolite profiles

BPTES exhibited significant effects on the metabolite profiles of all three cell types. However, its effect was most pronounced for cancer cells under hypoxia (Figure 2). Further, between the two cancer cell lines, the effect was more striking for MCF7 cells compared to MDA-MB231 cells. For example, BPTES altered 14 metabolite levels significantly for MCF7 cells (*p* < 0.05; 4 upregulated; 10 downregulated) under hypoxia, while only one was altered significantly (*p* < 0.05, upregulated) under normoxia. On the other hand, BPTES altered 7 metabolite levels significantly for MDA-MB231 cells (*p* < 0.05; 2 upregulated; 5 downregulated) under hypoxia, while only two metabolites were altered significantly (*p* < 0.05; 2 upregulated) under normoxia. BPTES induced up to 2-fold changes in metabolite levels (Table 1). For the non-cancerous cells, the response to BPTES was quite different; BPTES affected both normoxia and hypoxia cells significantly, albeit differently. For example, 7 metabolite levels were altered significantly (*p* < 0.05; 4 upregulated; 3 downregulated) under hypoxia, while under normoxia 10 metabolites were altered significantly (*p* < 0.05; 4 upregulated and 6 downregulated) (Figure 2; Table 1).

BPTES significantly affected the glycolysis pathway for both the cancer cell types under hypoxia (Figure 3). The effect, however, was different for the two cancer cells. Specifically, BPTES caused a significant reduction in glucose and increase in lactate levels for MCF7 cells, while the effect was opposite for MDA-MB231 cells. BPTES also affected a number of metabolites associated with the TCA cycle (Figure 4). In particular, it increased the levels of citrate for both MCF7 and MDA-MB231; however, the increase was significant (*p* < 0.05) only for MDA-MB231 cells.

### Hierarchical cluster analysis

**Figure 5:**
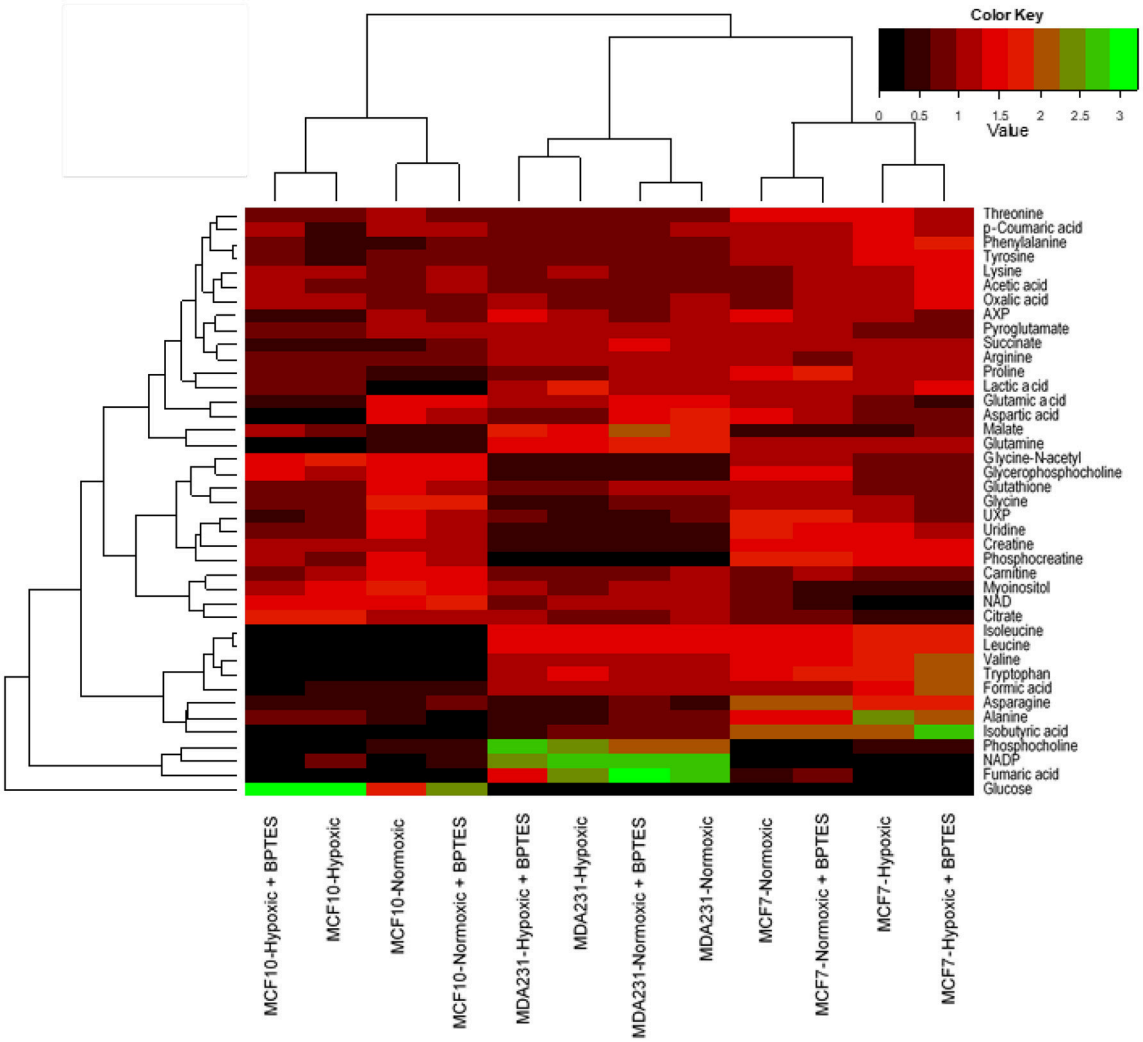
Results of hierarchical cluster analysis (HCA) of metabolic profiles in BPTES treated and untreated cells (MCF10A, MCF7, and MDA-MB231) under normoxic and hypoxic conditions. Relative NMR peak areas were used in the analysis (for interpretation of the references to color in the description of this figure in the text, the reader is referred to the Web version of this article).

For global visualization of the altered metabolite profiles the levels of the 41 metabolites from all groups of cells were combined and subjected to hierarchical cluster analysis (HCA). Figure 5 shows the dendrograms from HCA for all the three cell types under normoxia, hypoxia, and with and without treatment with BPTES. The metabolic phenotypes exhibited distinct clustering in HCA based on cell type as well as the effect of hypoxia and BPTES treatment. In particular, the clusters between cancerous and non-cancerous cells showed the largest distance in HCA, indicating that the difference in the metabolic profiles between the two cell types is most pronounced. The second largest distance observed was between the two cancer cell lines, MCF7 and MDA-MB231. Clusters of the same cells between normoxia and hypoxia cells exhibited the third largest distance. Finally, the cells that were treated with BPTES and those that were not treated were the least separated.

### Pearson correlations

**Figure 6:**
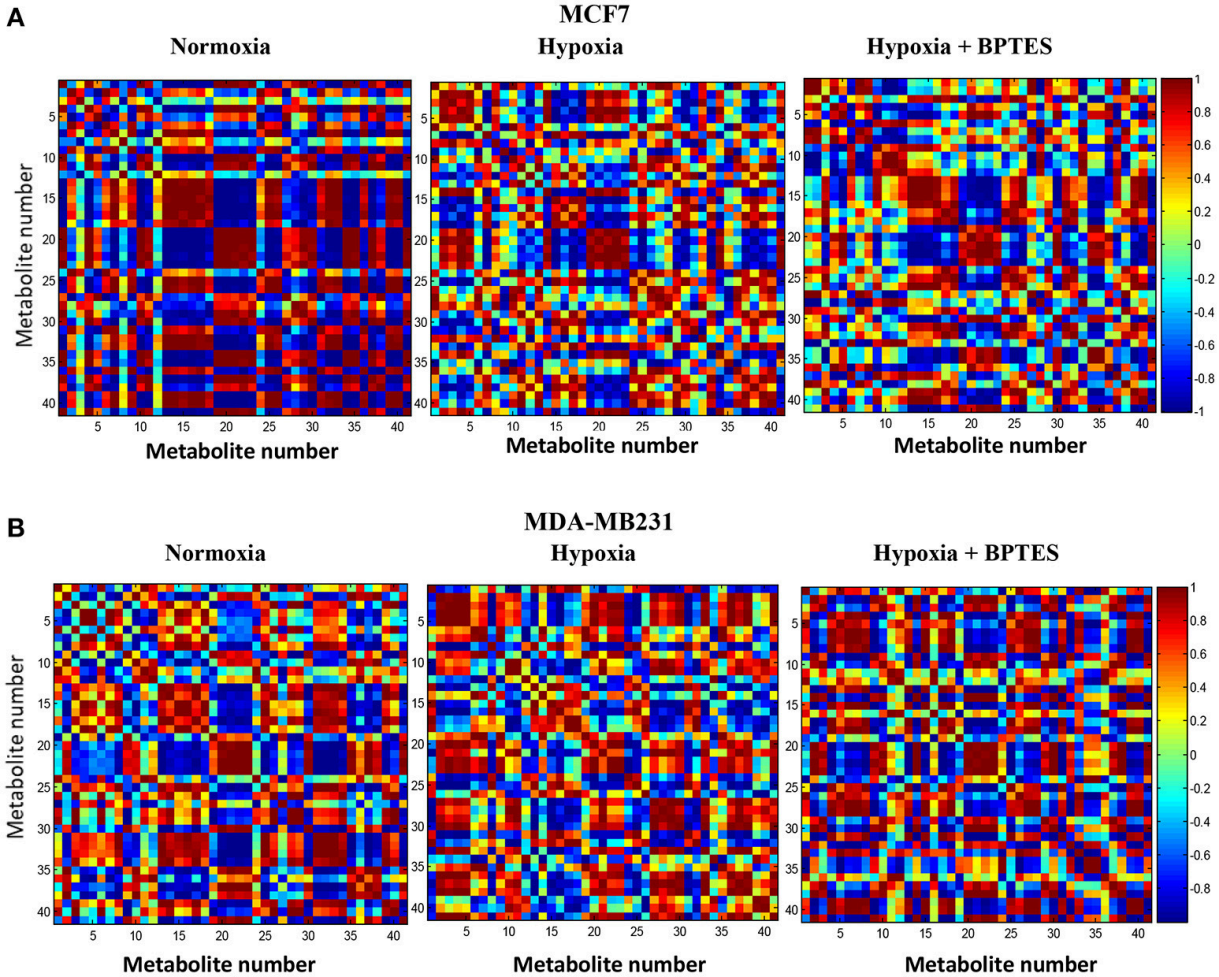
Pearson's correlations of the quantities of the 41 metabolites determined by NMR spectroscopy in human breast cancer cell lines **(A)** MCF7 and **(B)** MDA-MB231 under normoxia, hypoxia and hypoxia with BPTES treatment as indicated. The numbers for the metabolites used are as given in Table 1. Each square between any two metabolites in the 2D plots represents the magnitude of correlation between them. The vertical chart on the right indicates color code for the correlations. For example, red indicates a correlation of +1 and blue indicates a correlation of −1 (for interpretation of the references to color in the description of this figure in the text, the reader is referred to the Web version of this article).

Pearson correlations for the 41 metabolites from the two breast cancer cells, MCF7 and MDA-MB231, under normoxia, hypoxia and hypoxia with BPTES treatment are shown in Figure 6. The correlations for the non-cancerous cell line, MCF10A, are shown in the Supplementary Figure S1. Both hypoxia and BPTES altered a number of correlations significantly for both breast cancer cells. For instance, for MCF7 cells under hypoxia, glutamine is strongly positively correlated with lactate, 3-methyl-2-oxovalerate, citrate, pyroglutamate, isoleucine, leucine, lysine and valine, and strongly negatively correlated with glucose, glutamic acid, glutathione, glycine, glycerophosphocholine, phosphocholine, myoinositol, tryptophan, uridine, and fumaric acid. All these correlations were opposite after BPTES treatment. Similarly, for MDA-MB231 cells under hypoxia, glutamine is strongly positively correlated with lactate, AXP, carnitine, glycine and 3-methyl-2-oxovalerate and strongly negatively correlated with glucose, formate. All of these correlations were opposite in the BPTES treated cells.

## Discussion

This study focused on investigating the effects of BPTES on the metabolism in several breast cancer cell lines using a combination of 1D and isotope tagged 2D NMR-based metabolomics approaches to visualize the altered metabolite profiles. The cell studies were performed under both normoxia and hypoxia, with a major emphasis on the investigations of breast cancer cells under hypoxia. Cancer cell metabolism under hypoxia is considered to better mimic the *in vivo* environment, where proliferating tumors experience a reduced oxygen supply due to inadequate blood supply. It is well known that hypoxia leads to numerous consequences in invasive and metastatic cancers including a shift toward anaerobic glycolysis, away from the oxidative mitochondrial phosphorylation. Hypoxia increases dependence on glutamine for ATP synthesis (Fan et al., 2013) and also important for reductive metabolism for lipogenesis (Metallo et al., 2011). In the present study, the effect of hypoxia is reflected in significantly altered levels of metabolites for nearly half of the detected cell metabolites (Figures 2–4). The two breast cancer cell lines, MCF7 and MDA-MB231, showed vastly different metabolite levels relative to each other as well as to the control cells, even in the absence of BPTES treatment. Such differences arise from the fact that the two breast cancer cell lines exhibit distinct characteristics, clinically and pathologically (Rouzier et al., 2005; Neve et al., 2006; Ibrahim et al., 2009). While MCF7 cells express high levels of estrogen receptor and are dependent on estrogens for growth, MDA-MB231 cells are estrogen-independent and do not express estrogen receptor very highly (Thompson et al., 1988). In addition, MCF7 cell line represents luminal-like and MDA-MB231 cell line represents basal-like breast cancer. Importantly, the major metabolic differences between the two breast cancer cells observed in the current study are in accordance with a previous investigations using ^1^H NMR (Lefort et al., 2014). Metabolic changes in basal-like and luminal-like cancers have been investigated using high-resolution magic angle spinning NMR in xenografted primary human breast tumors (Moestue et al., 2010). Distinct metabolic profiles in the two xenograft models were identified in accordance with the differences in gene expression. In particular, choline metabolite concentrations differed significantly between the two subtypes.

BPTES treatment significantly altered the levels of a number of metabolites in both breast cancer cell lines, and such altered levels clearly indicate the cells' sensitivity to the glutaminase inhibitor BPTES (Figures 2, 5). The BPTES effect was distinctly different for the two cancer cell types: while 14 metabolites were altered significantly in MCF7 cell line, only 7 were altered significantly in MDA-MB231. Interestingly, other than phosphocholine, none of the significantly altered metabolites was common between the two cancer cell types. And even phosphocholine decreased in MCF7 while it increased in MDA-MB231 cells (Figures 2, 5). Such altered metabolite profiles due to BPTES can be understood based on the different metabolic pathway preferences between the two cell types. For example, the luminal-like MCF7 cells depend more strongly on glucose and, therefore, glycolysis dominates in these cells for energy. On the other hand, the basal like MDA-MB231 cells strongly depend on glutamine (Kung et al., 2011; Yizhak et al., 2014). Our results are in accordance with the recent metabolomics study by Terunuma et al., which exhibited a distinctly different metabolism for these two breast cancer cells and indicates that breast tumors with the same characteristics as MDA-MB231 cells exhibited poor prognosis (Terunuma et al., 2014). The differences in BPTES-induced metabolite levels for the two cancer cell types observed here also agree with the finding that resveratrol, a naturally occurring anticancer compound in red grapes and wine, exhibited vastly different metabolic activity toward these two cell type (Jäger et al., 2011). Further, interestingly, the major effect of BPTES on the metabolite profiles of MCF7 cells compared to MDA MB231 cells observed in our study is in accordance with the findings of a recent metabolomics study, which was focused on investigations of inhibitors that targeted pyruvate dehydrogenase kinase in the two types of breast cancer cells (Lefort et al., 2014).

Altered glucose metabolism is a major hallmark of cancer and accordingly, in the absence of BPTES treatment, glucose and lactic acid were altered significantly (*p* < 0.05) in both cancer cells relative to control cells (Table S2). The observed upregulation of glycolysis under hypoxia is consistent with earlier investigations (Weljie and Jirik, 2011) (Figure 2). However, interestingly, BPTES affected the glycolysis pathway only in MCF7 cell line; it caused a significant reduction of glucose and a concomitant increase of lactic acid (Figure 3). It is well known that MCF7 cells depend strongly on glucose and therefore glycolysis is the dominant pathway for this cell line (Kung et al., 2011). Inhibition of glutaminase activity by BPTES treatment potentially enhances the demand for energy, which leads to increased glycolysis. On the other hand, BPTES did not alter glycolysis for the MDA-MB231 cells, while the significantly decreased lactate may indicate that BPTES causes enhanced utilization of lactic acid. These results are in accordance with earlier findings that MDA-MB231 cells depend largely on external glutamine as source of energy (Kung et al., 2011); the glutamine dependence of MDA-MB231 cells is further supported from the fact that these cells have a higher expression of glutaminase (Jain et al., 2012). Because of their glutamine dependence, these cells are believed to be susceptible to glutamine targeted therapy (Kung et al., 2011). Thus, BPTES inhibition of glutamine metabolism, by triggering metabolic reprogramming involving an enhanced utilization of lactic acid, may account for the significantly decreased level of lactate (Figure 3). A previous study has shown that glutaminolysis adds to cellular production of lactate (Reitzer et al., 1979) and, therefore, the decreased lactate level in our study for MDA-MB231 cells also potentially represents a direct effect of inhibition of glutaminolysis by BPTES.

The Warburg effect in cancer is known to cause reduced TCA cycle activity due to the diversion of glucose to glycolysis instead of shuttling pyruvate for TCA cycle metabolism. Thus, the reduced level of citrate under hypoxia, in the absence of BPTES treatment, represents reduced TCA cycle metabolism for both MCF7 and MDA-MB231 cancer cell lines (Figure 4). Interestingly, BPTES treatment causes a significant increase in citrate levels for MDA-MB231, indicating that inhibition of glutaminase by BPTES blocks entry of glutamine to the TCA cycle and causes utilization of substrates through alternative pathways to fuel TCA cycle metabolism. Significant reduction of the levels of a number of metabolites including amino acids and nucleotides potentially indicates their enhanced utilization by the cells caused by inhibition of the glutamine supply to the TCA cycle metabolism (Figure 2). These results are also consistent with the reduction in the levels for a number of metabolites including amino acids due to BPTES treatment reported previously for glioma cells (Seltzer et al., 2010).

It is interesting to note that while the levels of a majority of metabolites decrease significantly after BPTES treatment for both cancer cell types, a number of organic acids including acetic acid, formic acid, and oxalic acid, as well as 3-methyl-2-oxovaleric acid (from isoleucine metabolism) increased in MCF7 cells, but not in MDA-MB231 cells (Figure 2). The reasons for such increased levels of organic acids are unknown; however, the results point to the increased activity of pathways associated with these metabolites as well as highlight the differences in pathway preferences in the two cell types. Further, while the level of phosphocholine decreased significantly in MCF7 cells, it increased significantly in MDA-MB231 cells due to BPTES treatment. An increased concentration of phosphocholine is in accordance with the increased demand for choline metabolites by MDA-MB231 cells due to their high proliferative characteristics compared to MCF7 cells, and is in accordance with earlier reports (Lefort et al., 2014).

**Figure 7:**
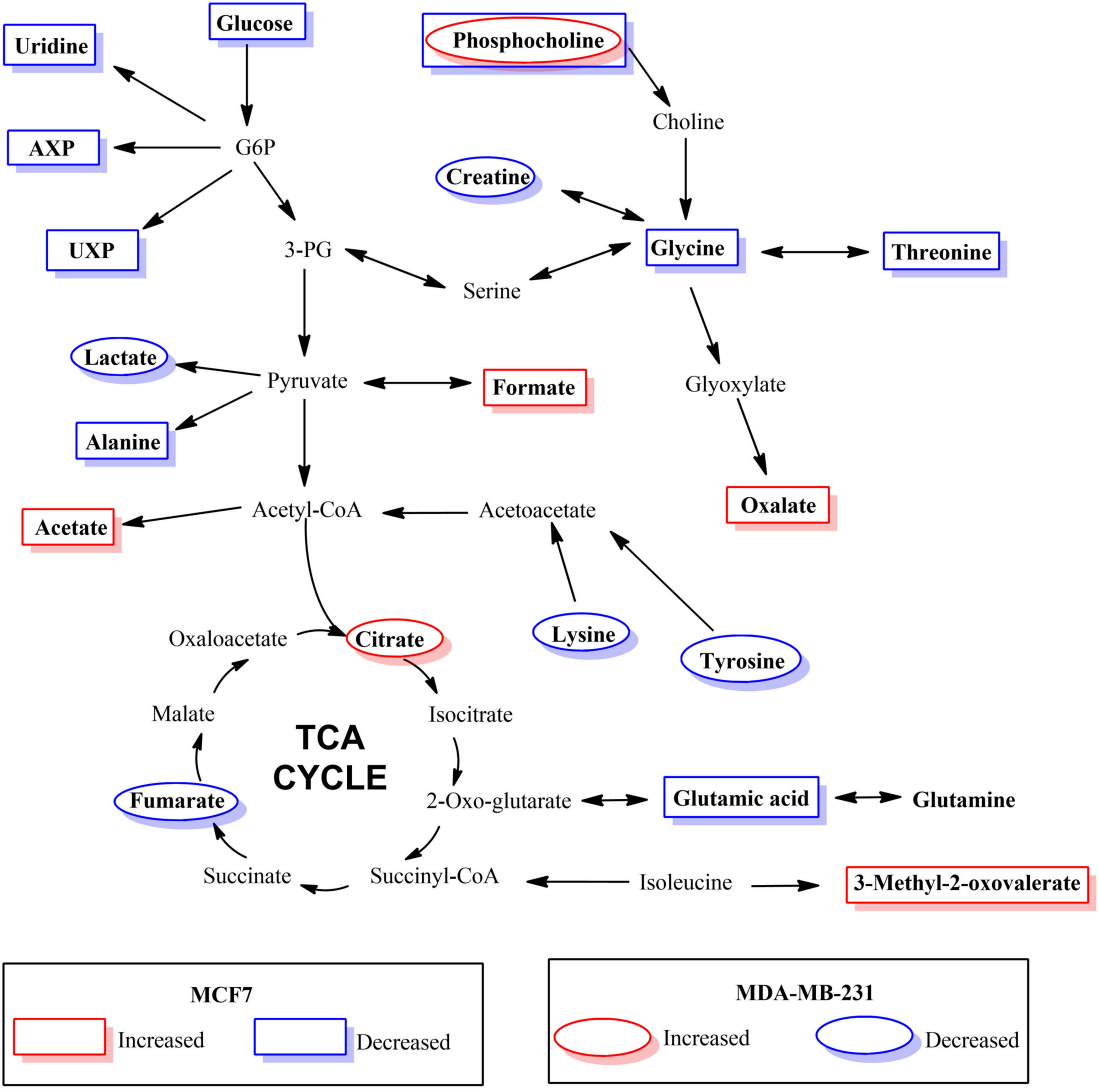
Depiction of metabolic pathways associated with metabolites that changed significantly due to BPTES induced metabolism in cancer cells under hypoxic conditions.

The results of hierarchical cluster analysis (HCA) indicate that the metabolic phenotypes exhibited distinct clustering based on cell type as well as the effect of hypoxia and BPTES treatment (Figure 5). As anticipated from the results of univariate analysis (Figure 3, Table 1), the cell phenotypes and hypoxia caused major effects on the metabolic profiles followed by a more subtle BPTES effect; cells treated with BPTES and those that were not treated formed the closest clusters in HCA. Similarly, Pearson correlations provide a global view of the metabolic perturbations, and showed large changes in the relationships of a number of metabolites as a result of stressors such as hypoxia and BPTES (Figure 6; Supplementary Figure S1). Such correlations in combination with the results of univariate analysis provide important clues to tracing altered metabolic pathways due to hypoxia or BPTES. From these results we can provide a visualization in Figure 7 of the overall effect of BPTES on the two cancer cell lines, which includes pathways associated with significantly altered metabolites due to BPTES treatment under hypoxic conditions.

In conclusion, investigations of BPTES induced metabolism changes in two breast cancer cell lines, MCF7 and MDA-MB231 were performed using a combination of advanced NMR-based metabolomics techniques and statistical analysis methods. These investigations focus on the BPTES inhibition under hypoxia, the condition that best mimics the proliferating cancer *in vivo*, and the resulting metabolite profiles showed dramatic changes relative to the same cells under normoxia or non-cancerous MCF10A cells. Numerous metabolites associated with many pathways including glutamine metabolism, glycolysis, TCA cycle and amino acids pathways were significantly altered in response to BPTES. However, the metabolic response was distinctly different for the two cancer cell types. This is likely due to the different genetic regulations in the two cell types, preferences to estrogen receptor and dependence of glucose or glutamine for proliferation. The distinct metabolite responses to treatment, apart from providing clues to the molecular basis of glutaminase inhibition by BPTES, may potentially provide avenues for evaluating BPTES response and monitoring treatment.

## Author contributions

GN performed experiments and data analysis and wrote paper; GB performed experiments and data analysis; JD performed cell culture under hypoxia, with and without BPTES treatment; HG performed statistical data analysis; DM performed cell culture; DH involved in data interpretation and writing of the paper; DR conceptualized the study design; performed data analysis and interpretation and paper writing.

### Conflict of interest statement

DR reports holding equity and an executive position at Matrix Bio, Inc. The other authors declare that the research was conducted in the absence of any commercial or financial relationships that couldbe construed as a potential conflict of interest.
