# Supplementary material for: A Metabolomics Study of BPTES Altered Metabolism in Human Breast Cancer Cell Lines
Source: Front Mol Biosci. 2018 May 15;5:49. doi: 10.3389/fmolb.2018.00049 (PMC5962734; doi:10.3389/fmolb.2018.00049)
Supplement: Supplementary file 1 [file Data_Sheet_1.doc]

**Supplemental information**

**A Metabolomics Study of BPTES Altered Metabolism in Human Breast Cancer Cell Lines**

G. A. Nagana Gowda^1^, Gregory A. Barding Jr.^1^, Jin Dai^1^, Haiwei Gu,^1^ Daciana H. Margineantu,^2^ David M. Hockenbery^2^ and Daniel Raftery^1,2,3,*^

*^1^Northwest Metabolomics Research Center, Department of Anesthesiology and Pain Medicine, and ^3^Department of Chemistry, University of Washington, Seattle, WA 98109, ^2^Fred Hutchinson Cancer Research Center, Seattle, WA 98109*

| **Table S1:** Number of replicates of the three cell types (MCF10A, MCF7 and MDA-MB231) used for investigations of BPTES induced metabolism using NMR spectroscopy | | | | |
| --- | --- | --- | --- | --- |
| Cell Type | Normoxic Cells | | Hypoxic Cells | |
|  | BPTES treated | Untreated Controls | BPTES treated | Untreated Controls |
|  | 3 | 3 | 3 | 3 |
| MCF10A | 3 | 3 | 3 | 3 |
| MCF7 | 3 | 3 | 3 | 3 |
| MDA-MB231 | 3 | 3 | 3 | 3 |

| **Table S2:** Metabolic differences among the three cell lines (MCF10A, MCF7 and MDA-MB231) under normoxic conditions. | | | | | | | | | |
| --- | --- | --- | --- | --- | --- | --- | --- | --- | --- |
|  | **MCF7 vs MCF10A** | | **MDA-MB231 vs MCF10A** | | **MDA-MB231 vs MCF7** | | **Fold change** | | |
|  | **P<0.05**  **( n=25)** | **q*** | **P<0.05**  **( n=29)** | **q*** | **P<0.05 (n=27)** | **q*** | **MCF7/ MCF10A** | **MDA-MB231/ MCF10A** | **MDA-MB231/ MCF7** |
| Acetic Acid | 0.9 | 0.90000 | 0.009 | 0.013680 | 0.08 | 0.1131 | 1 | 0.8 | 0.8 |
| Alanine | 0.3 | 0.34688 | 0.006 | 0.010364 | 0.3 | 0.3324 | 4.3 | 2.2 | 0.5 |
| Arginine | 0.06 | 0.08222 | 0.007 | 0.011083 | 0.03 | 0.0513 | 1.4 | 1.8 | 1.2 |
| Asparagine | 0.0005 | 0.00185 | 0.7 | 0.718919 | 0.0002 | 0.0021 | 3.3 | 1.1 | 0.3 |
| Aspartic acid | 0.6 | 0.65294 | 0.1 | 0.122581 | 0.1 | 0.1281 | 1 | 1.2 | 1.2 |
| AXP | 0.2 | 0.24667 | 0.8 | 0.800000 | 0.3 | 0.3324 | 1.2 | 1 | 0.9 |
| Carnitine | 0.0004 | 0.00185 | 0.001 | 0.002000 | 0.04 | 0.0631 | 0.5 | 0.6 | 1.4 |
| Citrate | 0.01 | 0.02056 | 0.2 | 0.237500 | 0.02 | 0.0373 | 0.6 | 0.9 | 1.5 |
| Creatine | 0.006 | 0.01480 | 0.0001 | 0.000345 | 0.00002 | 0.0004 | 1.2 | 0.5 | 0.4 |
| Formic acid | 0.02 | 0.03364 | 0.001 | 0.002000 | 0.5 | 0.5256 | 2.8 | 3 | 1.1 |
| Fumaric acid | NA | NA | NA | NA | 0.004 | 0.0109 | NA | NA | 4.8 |
| **Glucose** | **0.00003** | 0.00028 | **0.0001** | 0.000345 | **0.2** | 0.2485 | **0.1** | **0.1** | **1.6** |
| Glutamic acid | 0.01 | 0.02056 | 0.3 | 0.325714 | 0.02 | 0.0373 | 0.8 | 0.9 | 1.2 |
| Glutamine | 0.0009 | 0.00278 | 0.00002 | 0.000127 | 0.008 | 0.0173 | 2.9 | 4.6 | 1.6 |
| Glutathione | 0.01 | 0.02056 | 0.0003 | 0.000814 | 0.02 | 0.0373 | 0.8 | 0.6 | 0.8 |
| Glycerophosphocholine | 0.06 | 0.08222 | 0.0000005 | 0.000010 | 0.0005 | 0.0029 | 0.9 | 0.4 | 0.4 |
| Glycine | 0.05 | 0.07400 | 0.007 | 0.011083 | 0.007 | 0.0159 | 0.7 | 0.5 | 0.7 |
| Glycine-N-acetyl | 0.02 | 0.03364 | 0.0007 | 0.001663 | 0.002 | 0.0068 | 0.7 | 0.4 | 0.5 |
| 3-Methyl-2-oxovalerate | NA | NA | NA | NA | 0.03 | 0.0513 | 71.2 | 28.3 | 0.4 |
| Isoleucine | 0.000002 | 0.00004 | 0.000001 | 0.000010 | 0.3 | 0.3324 | 118.4 | 122.5 | 1 |
| **Lactic Acid** | **0.002** | 0.00529 | **0.01** | 0.014074 | **0.7** | 0.7000 | **3.8** | **3.5** | **0.9** |
| Leucine | 0.000001 | 0.00004 | 0.000001 | 0.000010 | 0.05 | 0.0759 | 44.4 | 48.4 | 1.1 |
| Lysine | 0.7 | 0.74000 | 0.3 | 0.325714 | 0.7 | 0.7000 | 1 | 0.9 | 1 |
| Malate | 0.8 | 0.82222 | 0.002 | 0.003800 | 0.004 | 0.0109 | 0.9 | 3.7 | 4.1 |
| Myoinositol | 0.0004 | 0.00185 | 0.001 | 0.002000 | 0.0009 | 0.0041 | 0.3 | 0.5 | 1.6 |
| NAD | 0.02 | 0.03364 | 0.3 | 0.325714 | 0.1 | 0.1281 | 0.6 | 0.9 | 1.4 |
| NADP | NA | NA | 0.00009 | 0.000345 | 0.001 | 0.0041 | NA | NA | NA |
| Oxalic acid | 0.04 | 0.06167 | 0.03 | 0.039310 | 0.3 | 0.3324 | 1.3 | 1.3 | 1.1 |
| p-coumaric acid | 0.3 | 0.34688 | 0.6 | 0.633333 | 0.5 | 0.5256 | 1.2 | 1.1 | 0.9 |
| Phenylalanine | 0.0005 | 0.00185 | 0.003 | 0.005429 | 0.002 | 0.0068 | 1.9 | 1.5 | 0.8 |
| Phosphocholine | 0.0009 | 0.00278 | 0.00006 | 0.000285 | 0.00001 | 0.0004 | 0.2 | 3.9 | 18 |
| Phosphocreatine | 0.09 | 0.11893 | 5E-08 | 0.000002 | 0.00006 | 0.0008 | 1.1 | 0.1 | 0.1 |
| Proline | 0.0002 | 0.00148 | 0.0006 | 0.001520 | 0.003 | 0.0095 | 3.4 | 2.2 | 0.7 |
| Pyroglutamate | 0.5 | 0.56061 | 0.02 | 0.027143 | 0.005 | 0.0128 | 1 | 1.1 | 1.1 |
| Succinate | 0.02 | 0.03364 | 0.0002 | 0.000585 | 0.1 | 0.1281 | 1.8 | 2.2 | 1.2 |
| Threonine | 0.0005 | 0.00185 | 0.09 | 0.114000 | 0.0004 | 0.0027 | 1.4 | 0.9 | 0.6 |
| Tryptophan | NA | NA | NA | NA | 0.08 | 0.1131 | NA | NA | 0.8 |
| Tyrosine | 0.002 | 0.00529 | 0.01 | 0.014074 | 0.006 | 0.0145 | 1.8 | 1.4 | 0.8 |
| Uridine | 0.03 | 0.04826 | 0.00005 | 0.000271 | 0.0004 | 0.0027 | 1.2 | 0.5 | 0.4 |
| UXP | 0.1 | 0.12759 | 0.0002 | 0.000585 | 0.001 | 0.0041 | 1.2 | 0.5 | 0.4 |
| Valine | 0.00002 | 0.00025 | 0.000004 | 0.000030 | 0.04 | 0.0631 | 11.2 | 9.7 | 0.9 |
| NA: Not available due to weak or missing peak in one or more groups of cells.  *Benjamini-Hochberg corrected p values | | | | | | | | | |

**MCF10A**

**Normoxia Hypoxia Hypoxia + BPTES**


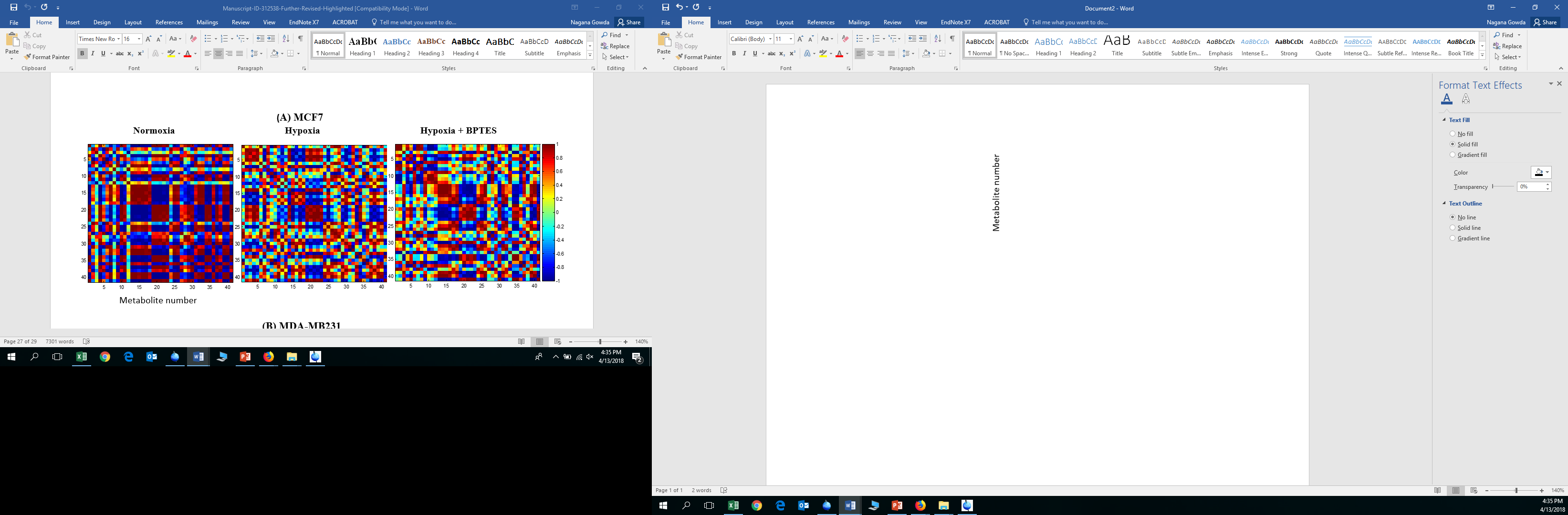


**Metabolite number**

**Metabolite number**

**Metabolite number**

**Supplementary Figure S1**. Pearson’s correlations of the quantities of the 41 metabolites determined by NMR spectroscopy in non-cancerous human breast cell line MCF10A under normoxia, hypoxia and hypoxia with BPTES treatment as indicated. The numbers for the metabolites used are as given in Table 1. Each square between any two metabolites in the 2D plots represents the magnitude of correlation between them. The vertical chart on the right indicates color code for correlations. For example, red indicates a correlation of +1 and blue indicates a correlation of -1. (For interpretation of the references to color in the description of this figure in the text, the reader is referred to the Web version of this article.)
